# Clinical Decision-Making and Multidisciplinary Management of Peristomal Pyoderma Gangrenosum in Stage IVB Rectal Cancer: A Case Report—Corticosteroid Response but Fatal Cancer Progression

**DOI:** 10.3390/reports9020194

**Published:** 2026-06-22

**Authors:** Hiroshi Tanabe, Mari Ogawa, Mari Kita, Takeshi Kotake

**Affiliations:** 1 Department of Dermatology, Tenri Hospital, Tenri 632-8552, Nara, Japan; 2 Department of Nursing, Tenri Hospital, Tenri 632-8552, Nara, Japan; maomari632@gmail.com (M.O.); kitamari@tenriyorozu.jp (M.K.); 3 Department of Medical Oncology, Tenri Hospital, Tenri 632-8552, Nara, Japan; tkkotake@gmail.com

**Keywords:** peristomal pyoderma gangrenosum, rectal cancer, panitumumab, pathergy, ultrasonic debridement

## Abstract

**Background and Clinical Significance:** Peristomal pyoderma gangrenosum (PPG) is a rare subtype of pyoderma gangrenosum, most commonly associated with inflammatory bowel disease or haematologic disorders. Its occurrence in patients with solid malignancies is uncommon. PPG in an oncologic setting poses diagnostic and therapeutic challenges because systemic immunosuppressive therapy, wound care, and ongoing chemotherapy must be carefully balanced; **Case Presentation:** We report the case of a Japanese man in his 50s with stage IVB rectal adenocarcinoma who developed rapidly progressive peristomal ulceration clinically consistent with PPG around a colostomy 12 weeks after initiation of panitumumab-containing systemic chemotherapy. The diagnosis was made on clinical grounds and was strongly supported by the clinical morphology, exclusion of major mimickers, and response to systemic corticosteroid therapy, although histopathological confirmation was not obtained. Because existing diagnostic criteria for pyoderma gangrenosum are not specifically designed for peristomal disease, they were used as supportive rather than definitive diagnostic tools. Skin biopsy was avoided due to the risk of pathergy at the peristomal site. Superficial cultures were not obtained because frequent cleansing and faecal contamination were likely to compromise diagnostic accuracy. To minimise mechanical pathergy, the stoma appliance was changed from a one-piece soft convex system to a two-piece flat system. Multidisciplinary management, including systemic corticosteroids, meticulous stoma care, and selective ultrasonic debridement, resulted in complete epithelialisation by Week 26. Chemotherapy was temporarily withheld during the active inflammatory phase and later resumed. Despite successful control of the peristomal ulceration, the patient died from progressive malignancy at Week 34; **Conclusions:** This case highlights the clinical challenge of balancing immunosuppressive therapy for clinically suspected PPG with ongoing oncologic treatment. Mechanical pathergy related to stoma appliance use was considered a more likely precipitating factor than chemotherapy alone, although panitumumab may have contributed to impaired cutaneous repair. Close collaboration among dermatologists, oncologists, surgeons, WOC nurses, and family caregivers is essential for multidisciplinary decision-making in complex oncologic settings.

## 1. Introduction and Clinical Significance

Pyoderma gangrenosum (PG) is an idiopathic neutrophilic inflammatory dermatosis. Peristomal pyoderma gangrenosum (PPG), a rare subtype, was first described by McGarity et al. in 1984 in three patients with Crohn’s disease [1]. PPG accounts for approximately 15% of all PG cases [2,3] and represents a severely painful complication that may arise following stoma creation. The reported incidence of PPG after abdominal surgery involving a stoma is approximately 0.6% [4]. Systemic diseases are present in about 86% of PPG cases, most commonly inflammatory bowel disease (IBD); however, the reported prevalence of PPG among patients with IBD varies widely (36–100%) [3,5]. Some studies suggest that advances in IBD therapy may influence the prevalence and potentially the pathogenesis of PPG [6]. PPG has been reported in approximately 7.4% of cases associated with solid tumours, although its occurrence in patients with colorectal cancer remains uncommon [5,7,8].

Regarding diagnosis, the Delphi consensus criteria [9] and the PARACELSUS score [10] for PG are frequently referenced, and their diagnostic performance has been evaluated; however, these tools include limited features specific to PPG and are not specifically designed for peristomal disease. PPG lacks disease-specific diagnostic biomarkers and is therefore a diagnosis of exclusion. Accurate diagnosis requires careful assessment of clinical morphology, microbiological cultures, histopathological findings, and response to immunomodulatory agents or systemic corticosteroids. Although skin biopsy and culture may assist in the evaluation of PPG, neither finding is disease-specific. In a systematic review of 335 PPG cases by Afifi et al., biopsy was performed in 174 cases (52%), whereas 22 cases (7%) were diagnosed without biopsy; cultures were obtained in 115 cases (34%) and most commonly yielded commensal cutaneous or intestinal flora [3]. Thus, cultures are not diagnostic of PPG, but they are useful for excluding primary or secondary infection and for supporting the absence of a clinically significant pathogen. However, in peristomal ulcers, culture results should be interpreted with caution because the ulcer bed may be affected by faecal contamination, colonization by enteric or skin flora, and repeated cleansing before sampling [11].

Histopathologically, PG is characterised by nonspecific neutrophilic abscesses and perifollicular neutrophilic inflammation. Differential diagnoses include other neutrophilic dermatoses, such as Sweet syndrome, infections, Behçet’s disease, and IBD-associated cutaneous manifestations. Pathergy is a characteristic clinical feature, presenting as pustules or ulceration induced by trauma or pressure. In the peristomal setting, mechanical factors such as frequent appliance changes and sustained pressure may trigger pathergic responses [1,12]. PPG-associated ulceration can compromise appliance adhesion, leading to leakage, delayed wound healing, and clinical deterioration [13]. PPG should be suspected in patients who develop rapidly progressive, painful peristomal ulcers that are refractory to standard management, particularly in those with underlying systemic disease such as IBD [3].

We report a case of PPG in a man in his 50s with stage IVB rectal cancer (cT4aN1M1) and multiple hepatic and pulmonary metastases, which developed 17 months after laparoscopic colostomy creation. This case represents a clinically informative example of PPG associated with rectal cancer in the absence of concomitant IBD. Although dermatology consultation is often initiated when PPG is suspected, optimal management requires a multidisciplinary approach. In addition to severe pain, patients face challenges related to stoma appliance management, prognosis, and oncologic treatment decisions. Close collaboration among oncology and surgical teams, wound, ostomy, and continence (WOC) nurses, and family caregivers is required. Because PPG lies at the intersection of medical, nursing, and caregiving domains, a comprehensive multidisciplinary strategy is essential for effective management [14].

## 2. Case Presentation

A Japanese man in his 50s was referred by the oncology department to our dermatology service for progressively worsening, painful peristomal skin ulcers. Seventy-eight weeks before his initial dermatology visit, the patient was diagnosed at our institution with unresectable stage IVB rectal cancer with multiple hepatic and pulmonary metastases. Histopathological and molecular analyses revealed RAS/BRAF wild-type, microsatellite instability-negative, HER2-negative, and UGT1A1 wild-type status. Palliative chemotherapy was selected, and a laparoscopic transverse colostomy was performed 76 weeks before the dermatology consultation. The patient had a history of anxiety and an allergy to oxaliplatin. He underwent sequential chemotherapy as follows: mFOLFOX6 plus bevacizumab (fluorouracil, leucovorin, oxaliplatin, and bevacizumab) from Week −75; fluorouracil plus bevacizumab from Weeks −53 to −35; FOLFIRI plus bevacizumab (fluorouracil, leucovorin, and irinotecan) from Weeks −33 to −22 because of tumour progression; and FOLFIRI plus panitumumab (Pmab) from Week −18 to the initial dermatology consultation (Table 1).

### 2.1. Progression of Peristomal Ulcers

Seventeen months after colostomy creation, five painful peristomal ulcers, each approximately fingertip-sized, developed. A wound, ostomy, and continence (WOC) nurse was consulted. Because the ulcers were suspected to be pressure-induced by the one-piece soft convex ostomy pouching system (CPbsf/VAbsf type; Dansac A/S, Fredensborg, Denmark), the appliance was switched to a two-piece flat ostomy pouching system using a New Image™ Flat FlexWear™ Skin Barrier (CPbh type; Hollister Incorporated, Libertyville, IL, USA). Treatment included application of a super-high-potency topical corticosteroid ointment combined with a silver-containing hydrofiber dressing, and use of a stretchable skin barrier. Despite six weeks of these interventions, the ulcers progressively enlarged, prompting dermatology referral (Table 2).

### 2.2. Dermatologic Findings at Initial Consultation

At the initial dermatology consultation, marked erythema and swelling were present throughout the ostomy barrier attachment area, accompanied by progressive enlargement of peristomal ulcers. Multiple painful ulcers with punched-out borders and undermined edges were observed, with adherent slough at the base. These characteristic findings were highly suggestive of PPG, and a strongly supported clinical diagnosis was made without skin biopsy due to the risk of pathergy. Because histopathologic confirmation and wound cultures were not obtained, the diagnosis was regarded as clinical rather than definitive.

**Figure 1:**
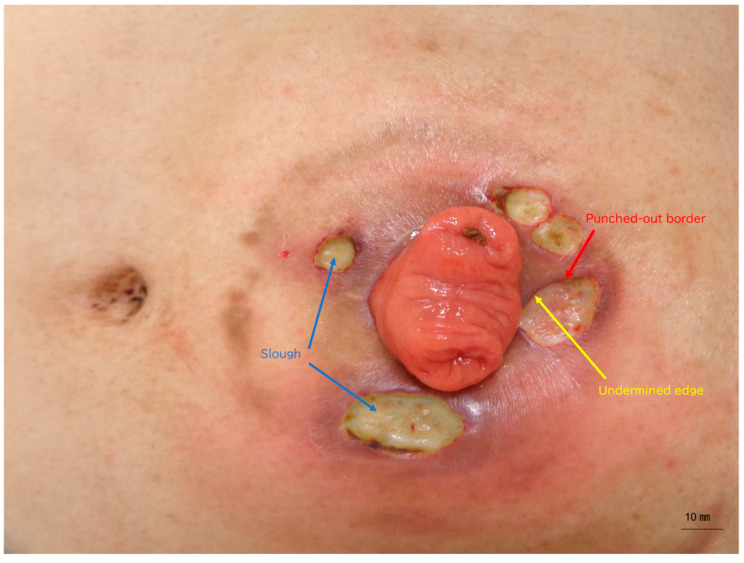
Clinical presentation at the initial dermatology consultation.

Stoma management was otherwise continued without major modification at that point. Topical corticosteroids were discontinued, and oral prednisolone was initiated at 20 mg/day (Figure 1).

Peristomal ulcers had developed over approximately 6 weeks prior to presentation. Despite stoma appliance modification and topical corticosteroid therapy, the ulcers progressively worsened. Five painful ulcers measuring 1–3 cm in diameter were observed around the colostomy site. The ulcers showed punched-out borders (red arrow), undermined edges (yellow arrow), and adherent slough at the base (blue arrow), findings consistent with but not specific for PPG. Direct microscopy of the surrounding skin was negative for fungi. Bacterial cultures were not obtained because frequent cleansing and faecal contamination were expected to compromise the interpretation of superficial swabs. The peristomal skin around the adhesive barrier was warm on palpation, without purulent discharge or systemic signs of infection.

### 2.3. Initial Laboratory Findings

Key laboratory results included: red blood cell count 3.80 × 10^6^/μL; haemoglobin 11.7 g/dL; white blood cell count 7.61 × 10^3^/μL (neutrophils 75.3%, lymphocytes 14.8%, monocytes 9.5%, eosinophils 0.1%); platelet count 188 × 10^3^/μL; C-reactive protein 1.79 mg/dL; AST 23 U/L; ALT 17 U/L; γ-GTP 57 U/L; blood urea nitrogen 25.8 mg/dL; and creatinine 0.4 mg/dL. Screening for infectious diseases was unremarkable.

### 2.4. Treatment Course

**Figure 2:**
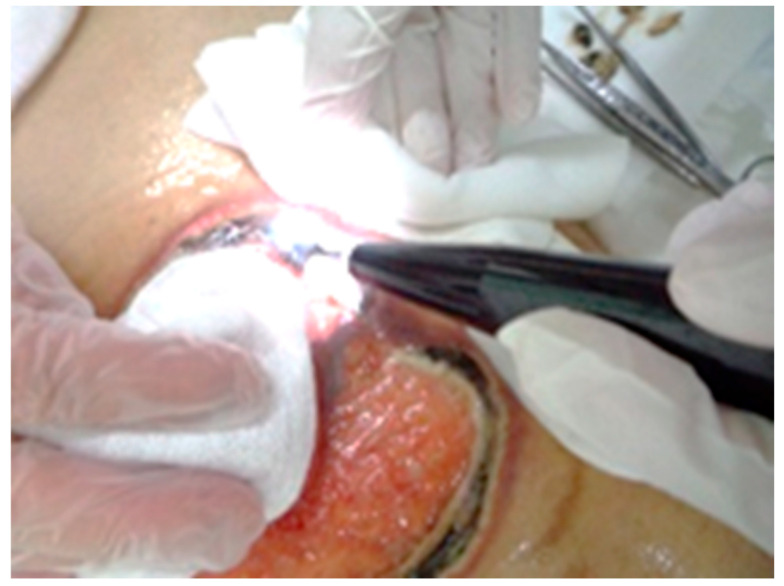
Selective ultrasonic debridement (Week 3–4).

One week after the initial dermatology consultation, peristomal erythema and swelling worsened, with increased warmth and further ulcer expansion. Necrotic tissue developed on the ulcer surfaces. As inflammation remained uncontrolled, prednisolone was increased from 20 mg/day to 40 mg/day (approximately 1 mg/kg/day), and Pmab was discontinued. The family was advised to increase the frequency of ostomy pouch changes. Three weeks after the initial consultation, the ulcers had expanded circumferentially around the stoma with extensive black necrosis. Severe pain and the risk of pathergy precluded conventional surgical or sharp debridement. However, the adherent necrotic tissue was contributing to exudate, appliance instability, and delayed wound-bed preparation; therefore, carefully limited selective debridement was considered necessary. A low-trauma ultrasonic debridement system (ULTRA Curette^®^, Gunze Medical, Osaka, Japan) was used to selectively remove necrotic tissue while preserving viable tissue and providing continuous irrigation (Figure 2) [15]. According to the manufacturer’s specifications, the device was operated at an ultrasonic frequency of 30 ± 2 kHz, with an amplitude of ≤75 μm, a power setting of 7, and a continuous irrigation flow rate of 20 mL/min. Due to concerns about impaired wound healing, all chemotherapy was discontinued. The ulcers extended beyond the ostomy barrier attachment area, resulting in excessive exudate and stool leakage. To stabilise the peristomal region, a convex insert was added to the two-piece flat system. Hospitalisation was recommended because of increasing difficulty with daily wound care and pain management, but the family opted for continuing home care, and the patient wished to maintain his work and home life.

Circumferential black necrotic tissue around the stoma was selectively debrided using an ultrasonic debridement system (ULTRA Curette^®^) following topical application of lidocaine jelly. To minimise pathergy, debridement was restricted to clearly nonviable necrotic tissue, and viable inflamed ulcer margins were avoided. Continuous irrigation was used during the procedure. Despite concerns regarding severe pain, the procedure was well tolerated, and the patient reported no pain during debridement.

**Figure 3:**
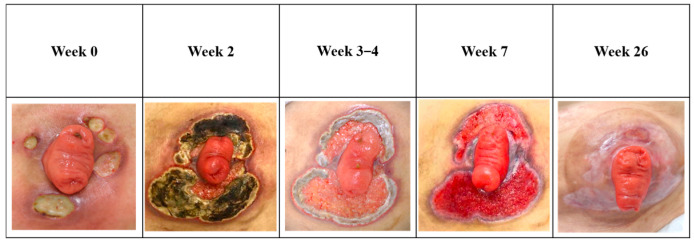
Clinical course following dermatology consultation.

Between Weeks 3 and 4 after dermatologic consultation, the necrotic tissue was removed, and healthy granulation tissue had begun to form. Although the ostomy appliance was unchanged, hydrocolloid dressings were added to manage persistent exudate. Topical trafermin (bFGF) was initiated to promote granulation, and prednisolone was tapered by 5 mg every two weeks. Imaging revealed progression of hepatic metastases. By Week 7, epithelialisation had begun from the ulcer margins. Wound care continued using silver-containing hydrofiber dressings, hydrocolloid dressings, and topical trafermin (30 μg per application), which was administered once daily for approximately 20 weeks (Figure 3).

Peristomal ulceration appeared six weeks before presentation. The stoma appliance was changed from a one-piece soft convex system (CPbsf/VAbsf type) to a two-piece flat system (CPbh type) due to suspected pressure-related injury; however, the ulcers continued to progress. At Week 0, a strongly supported clinical diagnosis of PPG was made, and oral prednisolone 20 mg/day was initiated. Due to worsening pain and erythema, the prednisolone dose was increased to 40 mg/day (approximately 1 mg/kg/day), and Pmab was withheld. By Week 2, circumferential black necrosis had developed. Selective ultrasonic debridement (Figure 2) was performed with care taken to minimise pathergy, followed by topical trafermin. Systemic chemotherapy was withheld during the active inflammatory phase and resumed at Week 7. Complete epithelialisation was achieved by Week 26, with minimal residual cribriform scarring. Representative images at Week 0, Week 2, Week 3–4, Week 7, and Week 26 illustrate progression from painful undermined ulcers to circumferential necrosis and subsequent epithelialisation. No images are reused from previous publications.

### 2.5. Chemotherapy Reinitiation and Outcomes

At Week 7, chemotherapy was resumed with FOLFIRI and continued for one month; silver-containing hydrofiber dressings were discontinued. From Weeks 15 to 20, the patient received trifluridine/tipiracil hydrochloride (TAS-102). At Week 20, CT imaging revealed further progression of hepatic metastases.

By Week 26, the peristomal ulcers had completely epithelialised, and ostomy appliance changes returned to the normal frequency of once or twice weekly. Hydrocolloid dressings were discontinued, and prednisolone was maintained at 2 mg/day. At Week 30, best supportive care was selected, and the patient passed away at Week 34.

### 2.6. Antibiotic Use and Microbiology

Minocycline (100 mg/day) was administered from Week −6 to Week 5 for infection prophylaxis. Despite approximately 6 weeks of therapy before dermatologic intervention, the peristomal ulcers progressed rapidly. Minocycline was continued for an additional 5 weeks after intervention and then discontinued, without apparent clinical benefit. Bacterial wound cultures were not obtained because of continual faecal contamination of the peristomal area and the limited specificity of superficial swabs in this setting. Direct potassium hydroxide microscopy was negative. No systemic signs of infection were observed.

## 3. Discussion

PPG remains a diagnosis of exclusion, and the present case should be regarded as a strongly supported clinical diagnosis rather than a histopathologically confirmed diagnosis. The diagnostic basis is summarised in Table 3, which maps the case to the Delphi consensus criteria, and in Table 4, which compares PPG with major mimickers of peristomal ulceration, consistent with previous reviews emphasising the broad differential diagnosis of PG [16]. In particular, infectious ulceration, irritant or contact dermatitis, pressure-induced ulceration, cutaneous metastasis, and drug-induced ulceration were considered less likely as primary diagnoses because of the clinical morphology, severe pain, rapid progression, suspected mechanical pathergy, lack of response to prolonged antibiotic therapy, and subsequent corticosteroid responsiveness.

The pathogenesis of PPG in this patient may be interpreted using a multi-hit framework, in which systemic predisposition, local pathergy, and treatment-related factors interact synergistically [11,17]. Advanced colorectal cancer may have contributed to a proinflammatory or immune-dysregulated systemic environment. Repeated appliance changes, continuous effluent exposure, and mechanical stress at the stoma site likely acted as persistent local triggers of pathergy. Prior exposure to anti-EGFR therapy was also considered as a possible contributory factor because such agents can impair epidermal repair and alter cutaneous inflammatory responses [18]. However, causality cannot be established from a single case, and the association with Pmab should be interpreted cautiously. In this case, Pmab may have contributed to impaired cutaneous repair within a multifactorial context, but mechanical pathergy and local peristomal factors were considered more direct precipitants of ulcer progression.

Previously reported cases of PPG associated with colorectal cancer are summarised in Table 5 [7,8,19,20,21]. Most reported cases occurred in patients without IBD and improved after systemic corticosteroid therapy. Compared with previous reports, the present case is notable because PPG was successfully controlled, whereas the underlying stage IVB rectal cancer progressed despite interruption and subsequent modification of oncologic therapy. This contrast highlights the therapeutic dilemma between achieving effective control of PPG through immunosuppression and maintaining cancer treatment in patients with advanced malignancy. The possible contribution of anti-EGFR therapy should therefore be interpreted as hypothesis-generating rather than causal.

A notable therapeutic aspect of this case was the use of selective ultrasonic debridement as a low-trauma alternative to sharp debridement, which may exacerbate PG or PPG through pathergy. The procedure was restricted to clearly nonviable black necrotic tissue, avoided the active undermined border, and was well tolerated without pain. Although evidence for ultrasonic debridement in PG or PPG remains limited, this case suggests that conservative, carefully selected debridement may be considered when adherent necrosis compromises wound-bed preparation or stoma appliance stability.

Early recognition of PPG is essential, as delayed diagnosis can lead to rapid ulcer progression, severe pain, and delayed initiation of appropriate immunosuppressive therapy. Because current diagnostic approaches rely heavily on clinical expertise and exclusion of alternative diagnoses, adjunctive tools to support early recognition may be beneficial [2]. Emerging advances in AI-based image analysis have shown promise in dermatologic pattern recognition [22] and may, in the future, assist in earlier triage and referral of suspected PPG cases; however, further validation is required before routine clinical use.

This case has several limitations. First, histopathological confirmation was not obtained due to the high risk of pathergy. Second, microbiological cultures were not performed, although clinical findings and treatment response strongly argued against infectious causes. Third, as a single case, the generalisability of the findings is limited.

This case has been partially reported in a Japanese nursing-focused journal, where the emphasis was placed on outpatient stoma care and nursing management [23]. In contrast, the present report provides a complementary medical perspective, focusing on diagnostic considerations, pathergy-related mechanisms, and multidisciplinary clinical decision-making. No figures, tables, or text have been reused from the previous publication.

## 4. Conclusions

We report a case of PPG that developed during chemotherapy in a man in his 50s with Stage IVB rectal cancer. The diagnosis was made clinically, without biopsy, based on the characteristic clinical morphology, rapid progression, exclusion of major mimickers, and response to systemic corticosteroid therapy. Systemic corticosteroids combined with stoma care and selective local wound management achieved complete epithelialisation. Chemotherapy was temporarily discontinued during the active inflammatory phase, and the patient ultimately died from progression of malignancy.

Although the precise aetiology of PPG in this case remains uncertain, mechanical pathergy related to stoma appliance use was considered a more likely precipitating factor than chemotherapy alone. Panitumumab may have contributed to impaired epidermal repair, but a causal relationship cannot be inferred from this single case. This case illustrates the clinical challenge of balancing effective PPG management with ongoing cancer treatment and emphasises the importance of close collaboration among oncologists, WOC nurses, surgeons, dermatologists, and family caregivers.

## Figures and Tables

**Table 1:** Patient clinical course from dermatology consultation (Week 0).

Week from Dermatology Consultation	Event
Week −78	First surgical consultation: Diagnosis of rectal cancer, Rs-Ra, cT4a N1 M1, Stage IVB.
Week −76	Laparoscopic transverse colostomy creation
Week −75 to −55	mFOLFOX6 + bevacizumab (BEV)
Week −53 to −35	5FU + bevacizumab (BEV)
Week −33 to −22	FOLFIRI + bevacizumab (BEV)
Week −19	First oncology consultation
Week −18 to 0	FOLFIRI + panitumumab (Pmab)
Week −6	WOC nurse intervention begins
Week 0	First dermatology consultation; strongly supported clinical diagnosis of PPG; prednisolone 20 mg/day started
Week 0	FOLFIRI + Pmab discontinued
Week 1	Ulcer worsened; Prednisolone increased to 40 mg/day
Week 2	Necrosis extended circumferentially around stoma
Week 3–4	Selective debridement using ULTRA Curette^®^; topical trafermin (30 μg/application) initiated; all chemotherapy discontinued.
Week 7–11	FOLFIRI restarted
Week 26	Complete epithelialisation achieved
Week 30	Transitioned to best supportive care
Week 34	Deceased

The table summarises the patient’s clinical course from initial cancer diagnosis to death. A strongly supported clinical diagnosis of PPG was made following ulcer development 17 months after laparoscopic colostomy creation and 12 weeks after initiation of Pmab. Following dermatology consultation (Week 0), systemic corticosteroid therapy was initiated. Chemotherapy was temporarily withheld during the active ulcer phase and later resumed. Complete epithelialisation of the peristomal ulcers was achieved by Week 26 following selective ultrasonic debridement and topical trafermin (30 μg per application, once daily for approximately 20 weeks). Owing to progressive malignancy, the patient was transitioned to best supportive care at Week 30 and died at Week 34.

**Table 2:** Management timeline from dermatology consultation (Week 0).

Week from Dermatology Consultation	Week −6	Week 0	Week 1	Week 3–4	Week 7	Week 26
Stoma appliance system	One-piece soft convex pouching system	Two-piece flat pouching system with mouldable skin barrier	Continued	Two-piece flat pouching system with convex barrier ring.	Continued	Continued
Wound dressing	Silver-impregnated hydrofiber dressing	Silver-impregnated hydrofiber dressing	Silver-impregnated hydrofiber dressing	Silver-impregnated hydrofiber dressing	Silver-impregnated hydrofiber dressing with hydrocolloid and trafermin (bFGF)	Hydrocolloid (dressing discontinued)
Steroid therapy	Topical corticosteroid applied under the wafer at each appliance change	Prednisolone 20 mg/day initiated	Prednisolone increased to 40 mg/day	Prednisolone 40 mg/day	Prednisolone tapered (30 → 25 → 20 → 15 mg/day every 1–2 weeks)	2 mg/day
Antibiotics	Minocycline 100 mg/day	Minocycline 100 mg/day	Minocycline 100 mg/day	Minocycline 100 mg/day + levofloxacin 500 mg/day	Discontinued	Discontinued
Analgesics	-	Loxoprofen sodium 60 mg/day	Loxoprofen sodium 120 mg/day	Loxoprofen sodium 120 mg/day + celecoxib 200 mg/day + tramadol/acetaminophen combination (2 tablets per dose)	Celecoxib 200 mg/day + tramadol/acetaminophen combination (2 tablets per dose)	Celecoxib 200 mg/day + tramadol/acetaminophen combination (2 tablets per dose)
Other notes	WOC nurse intervention initiated	Clinical diagnosis of PPG strongly supported; systemic corticosteroid therapy and multidisciplinary care involving dermatology, oncology, and WOC nursing initiated	Pmab discontinued	Selective ultrasonic debridement performed (ULTRA Curette^®^); chemotherapy discontinued	FOLFIRI restarted	Complete epithelialisation achieved; regular stoma care resumed

This table summarises changes in the ostomy appliance system, wound dressings, systemic corticosteroid therapy, antibiotics, and analgesics, together with key clinical and oncologic decision points, from 6 weeks before (Week −6) to 26 weeks after (Week 26) dermatology consultation. Representative clinical images are shown later in the manuscript. Time points are expressed as weeks relative to the dermatology consultation (Week 0).

**Table 3:** Mapping of the present case to the Delphi consensus criteria for pyoderma gangrenosum.

Criterion	Status	Evidence
MAJOR: Biopsy of ulcer edge showing neutrophilic infiltrate	Not performed (biopsy deferred due to pathergy risk and appliance considerations)	A skin biopsy was not performed because of the high risk of pathergy, severe pain, and concerns regarding pouch adhesion.
MINOR 1: Exclusion of infection	Yes	Cultures were not performed due to faecal contamination; KOH examination was negative; no systemic signs of infection; no response to antibiotics; rapid response to corticosteroids.
MINOR 2: Pathergy	Yes	Ulcer progression was temporally associated with repeated appliance changes, consistent with mechanical pathergy.
MINOR 3: History of IBD or inflammatory arthritis	No	The patient had metastatic rectal cancer; there was no history of inflammatory bowel disease or inflammatory arthritis.
MINOR 4: Papule, pustule, or vesicle ulcerating within 4 days	Unknown	Early transition from papule, pustule, or vesicle to ulceration within 4 days was not documented.
MINOR 5: Peripheral erythema with undermined, tender border	Yes	Violaceous, undermined, and exquisitely tender ulcer borders were observed on clinical examination and are shown in Figure 1.
MINOR 6: Multiple ulcers with ≥1 on an anterior lower leg	Not applicable (peristomal location)	Ulcers were confined to the peristomal region; no anterior lower-leg ulcer was present.
MINOR 7: Cribriform (“wrinkled paper”) scarring at healed sites	Yes	Healing resulted in epithelialisation with linear to cribriform (“wrinkled paper”) scarring observed at Week 26.
MINOR 8: Decrease in ulcer size within 1 month of immunosuppression	Yes	Ulcer size reduction was observed within 4 weeks after initiation of systemic corticosteroid therapy.
Total minor criteria met (out of 8)	5/8 (criteria 1, 2, 5, 7, and 8)	Five of eight minor criteria were fulfilled, supporting PPG as the most likely clinical diagnosis.

This table summarises the Delphi consensus criteria and their applicability to the present case. Although a biopsy was not performed, multiple minor criteria were fulfilled, supporting PPG as the most likely clinical diagnosis.

**Table 4:** Differential diagnosis of peristomal ulceration and basis for a strongly supported clinical diagnosis of PPG.

**Condition**	**Key Features**	**Findings in This Case**	**Conclusion**
Infectious ulcer (bacterial, fungal)	Purulence; positive cultures; systemic signs of infection; response to antibiotics	No purulence; cultures not performed due to faecal contamination; no systemic signs of infection; no response to antibiotics	Unlikely
Irritant/contact dermatitis	Erythema with erosion; limited to the appliance contact area; improves with appliance adjustment	Ulcers extended beyond the appliance area; progressive worsening despite appliance modification	Unlikely
Pressure-induced ulcer	Localised ulcer at pressure points; improves after pressure relief	Ulcers worsened despite appliance modification; rapid progression	Unlikely
Malignancy (cutaneous metastasis)	Nodular or infiltrative lesions; atypical cells on histology	No nodular lesions; rapid response to corticosteroids; no evidence of cutaneous metastasis	Unlikely
Drug-induced ulcer (e.g., anti-EGFR therapy)	Acneiform eruption; paronychia; delayed wound healing	No typical acneiform eruption; morphology was more consistent with PPG. A contributory effect of panitumumab (Pmab) on cutaneous repair cannot be excluded, but causality was not established.	Unlikely
Peristomal pyoderma gangrenosum (PPG)	Painful ulcers; undermined borders; rapid progression; pathergy; response to corticosteroids	Severe pain; rapid progression; circumferential necrosis; suspected pathergy associated with appliance changes; marked response to corticosteroids	Most likely

This table summarises the alternative diagnoses considered for the patient’s peristomal ulcers, together with the relevant clinical features, investigations, and rationale for exclusion. Collectively, these findings support PPG as the most likely clinical diagnosis over infectious, ischaemic, and other inflammatory causes.

**Table 5:** Previously reported cases of PPG associated with colorectal cancer.

Author (Year)	Cancer Type	IBD (Yes/No)	Trigger/Context	PPG Treatment	Outcome
Sakai et al. (2006) [19]	Rectal adenocarcinoma	No	Post-stoma placement	Systemic corticosteroids	Improved
Miguel-Gómez et al. (2015) [7]	Colon adenocarcinoma	No	Postoperative	Systemic corticosteroids	Improved
Olmedo-Martín et al. (2016) [8]	Rectal adenocarcinoma	Yes	Crohn’s disease	Systemic corticosteroids	Improved
Villalba Ferrer et al. (2020) [20]	Colorectal carcinoma	No	Chemotherapy-associated	Systemic corticosteroids; chemotherapy withheld	Improved
Present case	Rectal adenocarcinoma (Stage IVB)	No	Stoma trauma; anti-EGFR therapy contribution unproven	Systemic corticosteroids plus selective ultrasonic debridement	Ulcers healed; malignancy progressed

This table summarises published cases of PPG associated with colorectal cancer and includes the present case for comparison. To avoid potential duplication, only the earliest publication was included when overlapping cases were identified.

## Data Availability

The data presented in this case report are available from the corresponding author upon reasonable request, subject to patient privacy and ethical restrictions.

## Abbreviations

The following abbreviations are used in this manuscript:

PPG	Peristomal pyoderma gangrenosum
Pmab	Panitumumab
bFGF	Basic fibroblast growth factor
IBD	Inflammatory bowel disease
FOLFIRI	Folinic acid, fluorouracil, and irinotecan
BEV	Bevacizumab
EGFR	Epidermal growth factor receptor
WOC	Wound, ostomy, and continence
